# Herbivory legacy modifies leaf economic spectrum and drought tolerance in two tree species

**DOI:** 10.1007/s00442-025-05678-4

**Published:** 2025-02-26

**Authors:** Guillermo G. Gordaliza, José Carlos Miranda García-Rovés, Rosana López, Ismael Aranda, Luis Gil, Ramón Perea, Jesús Rodríguez-Calcerrada

**Affiliations:** 1 Departamento de Sistemas y Recursos Naturales, Escuela Técnica Superior de Ingenieros de Montes, Universidad Politécnica de Madrid, 28040 Madrid, Spain; 2 Instituto de Ciencias Forestales (ICIFOR-INIA), CSIC, Carretera de La Coruña K.M. 7.5, 28040 Madrid, Spain

**Keywords:** Multiple stress, Stress legacy, Ungulate browsing, Water relations, Recruitment

## Abstract

The concurring effect of herbivory by wild ungulates and drought events is experiencing a notable increase in Mediterranean and temperate forests. While many studies have addressed the influence of drought on plant susceptibility to herbivory, it appears crucial to comprehend the impact of prolonged browsing on the physiological response of plants to increasing water deficit. To this end, we analyzed the effect of long-term recurrent herbivory by ungulates on physiological, biochemical, anatomical and morphological variables of *Ilex aquifolium* and *Fagus sylvatica* saplings during the growing seasons of 2018 and 2019 in a mixed sub-Mediterranean forest. We compared plants growing within an exclosure fence since 2006 (unbrowsed) with plants growing outside (browsed) that were also fenced during the study to investigate herbivory legacy. Twelve years of herbivory pressure modified significantly plant functional performance. Independently of the species, browsed plants showed higher root-to-shoot ratio, stem cross-sectional area-to-leaf area ratio, predawn leaf water potential, leaf nitrogen concentration and leaf gas exchange rates than unbrowsed plants. Moreover, browsed plants had lower leaf bulk modulus of elasticity, and higher osmotic potential at full turgor and turgor loss point. Thus, herbivory modified the leaf economic spectrum towards a more resource-acquisitive and less water stress tolerant type. These results suggest that, once browsing has subsided, plants continue to reflect some legacy effects that make them more vulnerable to further abiotic and biotic stresses, which has implications for forest regeneration.

**Supplementary Information:**

The online version contains supplementary material available at 10.1007/s00442-025-05678-4.

## Introduction

The significant increase in the population of wild ungulates in the Northern Hemisphere over the last decades (Carpio et al. 2017; Valente et al. 2020) is triggering unprecedented levels of herbivory (Leland Russell et al. 2001; Côté et al. 2004; Morales-Molino et al. 2019). Ungulate browsing has become a major factor hindering natural regeneration in many temperate, boreal and Mediterranean forests (Zamora et al. 2001; Côté et al. 2004; Pulido and Díaz 2005; Pellerin et al. 2010; López-Sánchez et al. 2019). After browsing, plants suffer morphological alterations, such as reduced height and foliage density often leading to significant growth loss (Drexhage and Colin 2003). However, in some cases, plants are able to recover and overcome the effects of browsing (Staver and Bond 2014). As an adaptation to herbivory, plants have developed a series of mechanisms of tolerance and/or resistance, such as high root–shoot ratio, increased leaf photosynthesis, rapid resource allocation from root to shoot upon browsing (Strauss and Agrawal 1999; Tiffin 2000), or leaf spinescence, pubescence and sclerophylly (Hanley et al. 2007). Due to differences in herbivory susceptibility, repeated browsing can cause changes in the structure and composition of woody plant communities, as the most palatable and/or least adapted species reduce their abundance (Gill and Beardall 2001; Rooney and Waller 2003; Perea et al. 2014; Lecomte et al. 2024).

Another key factor affecting forest dynamics in southern parts of Europe and western North America is drought-induced tree mortality and forest die-off, related to an increase in the frequency, duration, and/or severity of drought events (Hammond et al. 2022). Depending on the growth stage of the plant, a deficit in water supply affects a broad set of morphological, physiological and biochemical processes, such as growth and productivity, water relations, mineral uptake and assimilation, and light harvesting and carbon fixation (Farooq et al. 2012). Thus, to avoid lethal drought stress, plants modulate plant biomass allocation and root architecture (Malamy 2005), reduce transpiration, increase water use efficiency and adjust the concentrations of osmolytes to, collectively, maximize soil foraging for water, minimize water loss, and extend carbon gain in dehydrating leaves (Poorter and Markesteijn 2008).

In natural conditions, plants must face multiple co-occurring stresses. Plant acclimation to simultaneous stresses, e.g., drought and shade (Aranda et al. 2001, 2002), heat and drought (Rennenberg et al. 2006; Zandalinas et al. 2018), shade and herbivory (Blundell and Peart 2001) or drought and herbivory (Bansal 2015; Gieger and Thomas 2002) often involves phenotypic changes that are not the sum of those observed under each stress separately. Multiple studies have addressed the way drought stress affects plant susceptibility to herbivores, especially insects (White 1984; Huberty and Denno 2004; Gutbrodt et al. 2011; Gely et al. 2020). Much less effort has been made to understand how herbivory, especially long-term, sustained browsing, affects drought resistance in plants (Johnston et al. 2007; Pittermann et al. 2014; Carvajal Acosta et al. 2023). That is, does browsing affect the capacity of the plants to avoid or tolerate desiccation stress?

The co-occurrence of herbivory and drought stress often has antagonistic effects on plants, since herbivory tends to increase the need for carbon, while drought stress decreases carbon gain (Taiz and Zeiger 2010). Moreover, the need to defend from ungulates by producing secondary metabolites may divert carbon and energy for osmotic adjustment, which is a common mechanism of drought tolerance (Munns 1988). Repeated browsing of shoots may also alter hydraulic architecture and reduce xylem area-specific conductivity, if sustained reductions in carbon gain affect xylem development (Pittermann et al. 2014; Ueda et al. 2024; Leonard et al. 2024). However, the effect of sustained herbivory over time on drought response may not necessarily be negative (Bansal et al. 2013). Browsing during the vegetative period may reduce leaf area and plant transpiration (Peláez et al. 2025), an effect that may be partly offset by compensatory growth, and increase leaf-specific hydraulic conductivity and gas exchange rates (Johnston et al. 2007). Furthermore, browsing can enhance biomass partitioning to the roots, which may favor drought avoidance through the formation of lateral roots and/or a larger taproot (Drexhage and Colin 2003). It is important to study the joint effect of long-term herbivory pressure and soil water deficit, two main stressors in Mediterranean and sub-Mediterranean forests, to gain a deeper understanding of tree acclimation responses and their consequences for tree recruitment, and forest structure and function (Didion et al. 2009; Nuttle et al. 2011; Frerker et al. 2014; Pendergast et al. 2015).

This study aims to uncover how herbivory legacy affects drought stress intensity and tolerance. To this end, during the growing seasons of 2018 and 2019, we examined leaf water relations, photosynthetic, biochemical, anatomical and morphological traits of *Ilex aquifolium* and *Fagus sylvatica* saplings naturally regenerated in a sub-Mediterranean mixed broadleaf forest under high vs. null ungulate pressure since 2006. We compared both species because they are predominant in European broadleaf forests and because they differ in leaf habit and lifespan, and probably in herbivory sensitivity (Salleo et al. 1997; Retuerto et al. 2006; Gossner et al. 2014; Rita et al. 2015; Leuschner 2020; Perea et al. 2020). The evergreen leaf habit of *I. aquifolium* (hereafter *Ilex*) implies a greater risk of browsing than the deciduous leaf habit of *F. sylvatica* (herafter *Fagus*); however, *Ilex* has greater resistance to herbivory due to its thick, hard and spiny leaves, and its significant capacity to resprout when the entire shoot is cut or eaten (Mountford and Peterken 2003; Perea et al. 2020). Both species are very tolerant to shade and sensitive to water stress, although the sclerophyll leaf of *Ilex* typically confers greater ability to avoid water stress (van Hees 1997; Niinemets and Valladares 2006). Moreover, there is a great difference between both species in their growth rates, with *Fagus* having 10 times higher stem annual radial growth in this study area (Rodríguez-Calcerrada et al. 2019). We hypothesized that (1) sustained browsing would increase the root-to-shoot biomass ratio and that, for this reason, (2) browsed saplings would experience less water stress during summer drought than protected, unbrowsed saplings, and so (3) would be less drought tolerant. In a fourth hypothesis, we expected that species would differ in response to herbivory. The coriaceous, spiny leaves of *Ilex* would be a constitutive defence that would reflect limited herbivory-induced plasticity, as compared to faster growing, less sclerophyll *Fagus* (Sun et al. 2023).

## Materials and methods

### Study area and study species

This study was conducted in a 125-ha mixed broadleaf forest located in the Ayllon mountains, Central Spain, (3°30’W, 41°07’N), at 1250–1500 m above sea level. The forest has six types of pure to mixed stands dominated by *Fagus sylvatica* L, *Quercus petraea* (Matts.) Liebl. and *Quercus pyrenaica* Willd. In addition, *Ilex aquifolium* L. is widely found in the understory of all stands, especially those of *Fagus*. The forest is part of the World Heritage Primeval Beech Forests of the Carpathians and Other Regions of Europe and represents one of the southernmost European populations of several temperate tree species (e.g., *F. sylvatica, I. aquifolium*, *Prunus avium* L. and *Q. petraea*). Soils are formed from mica schists and mica gneiss substrates. Climate is sub-Mediterranean, with 9.9 °C mean annual temperature and 879 mm annual rainfall—average data from 1994 to 2021 recorded in a meteorological station located in the forest. Both variables exhibit a strong seasonality: mean monthly minimum (0.5 °C) and maximum (24.0 °C) temperatures are reached in January and July, respectively, and a dry period of 131 mm rainfall (on average) extends from June through September. As other marginal populations, the stand is potentially sensitive to warming-induced drought stress (Rozas et al. 2015).

Roe deer (*Capreolus capreolus* L.) and cattle are the main large herbivores in the study area. High habitat quality and changes in land use, mainly increased forest cover and tree density, have led to an increase in the density of roe deer over the last decades (Aragon et al. 1995; Tellería and Virgós 1997), with a current density of 5.56 individuals km^−2^ in the UTM square of Ayllon-Guadalajara (Acevedo et al. 2010), near the study forest. Deer populations have increased in the whole study area similarly to many other areas in Europe (Burbaite and Csányi 2009) and are considered overabundant in many areas (Carpio et al. 2021). The abundance of wild boar (*Sus scrofa L.*) has also increased in recent decades, with an estimated density of 1.0–1.6 individuals km^−2^ in the study area (Acevedo et al. 2014). Cattle presence is frequent, particularly in summer, when temperatures in surrounding grasslands are too high.

The high ungulate pressure and summer drought severity in this forest affect the regeneration of *Ilex* and *Fagus*. Despite they are not preferred food sources in the forest (Perea et al. 2020) it is rare to find saplings of any of the two species that are not intensively browsed.

### Study design

In January 2018, five plants per species were selected inside a 0.5 ha ungulate exclosure (hereafter, unbrowsed plants) and outside (hereafter, browsed plants). The ungulate exclosure was established in 2006 using 150-cm high wire fences. Importantly, the plants located outside the ungulate exclosure were protected individually during the entire study—2018 and 2019—by metal mesh (31 mm mesh size) to ensure that plants were not damaged by the action of ungulates. This way we were sure of addressing long-term browsing effects on plant traits, without the uncontrolled, variable impact of ungulates during the years of study. Maximum distance between individuals was 68.0 ± 3.7 m (mean ± SE). Plant light availability was assessed with one hemispherical photo (Nikon digital camera with a sigma 8-mm fisheye lens) taken over each plant further analyzed with Hemiview 2.1 Canopy Analysis Software (Delta-T Devices Ltd, Cambrigde, UK). The global site factor (i.e., the proportion of light in the understory relative to an open site), ranged between 16 and 28%. No statistically significant differences were found between browsed and unbrowsed plants for this variable at *P* < 0.1. Plants selected outside the fence showed symptoms of heavy browsing, with these plants having most of the shoots eaten by ungulates. Thus, mean plant height and stem base diameter were higher for unbrowsed than browsed plants at *P* < 0.01: 28.7 ± 1.4 vs. 20.9 ± 1.2 mm diameter and 153.1 ± 6.5 vs. 72.9 ± 5.7 cm height, respectively. An analysis of growth rings at the stem base in harvested plants indicated that mean age was 21.7 ± 1.9 years in browsed plants and 17.6 ± 1.8 in unbrowsed plants (*P* > 0.10).

Measurements were taken during the growth periods of 2018 and 2019. Photosynthetic and biochemical variables were measured three times during each growing season, in late spring, midsummer and late summer, with the intention of capturing the progressive increase in drought intensity. Pressure–volume (PV) curves were constructed once a year, in late summer. Finally, plant anatomical and morphological variables were measured at the end of the experiment, after the harvest of the studied plants in October 2019.

### Leaf water potential

Leaf water potential (Ψ) was measured with a Pressure Chamber (Model 1000, PMS, Instrument Company, USA) using the standard protocol established by Scholander et al. (1965). Leaf water potential was measured at dawn (Ψ_pd_, before 7:00 am local time), when it is maximum and indicative of the soil water accessible to the roots, and at mid-day (Ψ_md_, from 13:00 to 16:00 pm), when water potential reaches the minimum diurnal value.

### Photosynthetic parameters

An infrared gas analysis (IRGA) system (Li-6400; Li-Cor Inc., Lincoln, NE, USA), coupled to a broadleaf chamber (LCF-40 chamber LI-COR Inc., Lincoln, NE, USA) was used to estimate net photosynthetic rate (A_area_), stomatal conductance to water vapor (g_s_), intercellular CO_2_ concentration (C_i_) and electron transport rate (ETR). Intrinsic water-use efficiency (iWUE), a measure of carbon assimilation per unit of water used, was calculated as the ratio of A_area_ to g_s_. The ETR was calculated as: actual quantum yield of electron transport through photosystem II (Φ_PSII_) × photosynthetic photon flux density (PPFD) × 0.5 (considering excitation energy divides equally between two photosystems) × 0.84 (a common leaf absorbance coefficient for C3 plants; Flexas et al. 1999). Φ_PSII_ was calculated according to the equations given in Maxwell and Johnson (2000). Measurements were taken at 400 ppm air CO_2_ concentration, 25.0 °C air temperature, ambient air relative humidity, 300 µmol s^−1^ air inlet flow rate and 1200 m^2^ s^−1^ PPFD, in one healthy leaf per individual selected from the upper outer part of the canopy. This leaf was representative of the individual, given the small size of plants and high morphological homogeneity of foliage. The measurements were made between 10:30 and 14:00 h, alternating measurements between species and at least twice between treatments. These leaves were used to calculate the Leaf Mass per Area (LMA) and from it, the net photosynthetic rate per unit of leaf dry mass (A_mass_).

### Biochemical measurements

For the analysis of C and N concentrations ([C] and [N]), and C and N isotope composition (δ^13^C and δ^15^N), we used the same leaves in which photosynthetic measurements had previously been made. These samples were sent to Department of Plant Sciences (University of California, Davis, California, USA) for their measurement with an elemental analyzer. Approximately 4 mg of samples oven-dried at 60 °C for 72 h were prepared following the protocol established by the UC Davis Stable Isotope Facility.

### Pressure–volume curves

Pressure–volume curves were constructed using one healthy leaf per plant taken close to the leaves used for photosynthetic and biochemical measurements. They were collected at dawn in plastic bags and their petioles immersed in deionized water for rehydration during the following hours. Weight and water potential were measured periodically as the leaves gradually dried, until at least five data points were recorded after the turgor loss point, with a total of 11–19 measurements per curve. Leaves were then oven-dried at 70 °C for 72 h to calculate leaf dry weight.

Relative water content at the turgor loss point (RWC), relative symplastic and apoplastic water contents (R_s_ and R_a_, respectively), osmotic potential at full turgor (π_100_), osmotic potential at the turgor loss point (π_0_) and dry weight-to-turgor weight ratio (D_W_/T_W_) were derived from the PV curves as described by Turner (1988). The maximum bulk modulus of elasticity (ε_max_) was calculated following Robichaux (1984).

### Biomass partitioning

At the end of the study, aboveground biomass was harvested using pruning shears. Root biomass was measured only for *Fagus*. The enormous root system of *Ilex* made its excavation too time-consuming and laborious, with the additional risk of having inaccurate data. Roots were collected manually using a mattock tool (38.1 cm), for preparing the ground, and a set of chisels (8–11.5–22 mm) for the individualized extraction of roots. Root depth (RD) was measured in situ with a measuring tape. In the laboratory, roots were washed thoroughly with tap water through a 0.5 mm screen sieve. Root and shoot (leaves and stem) dry mass (RB and SB, respectively) were determined after drying the plant material in an oven at 72 °C for 72–96 h.

The root-to-shoot ratio (R/S) was calculated as the ratio of root dry weight to aboveground dry weight. The total leaf area (LA) was estimated as the product of the total dry weight and the ratio between the leaf area and dry weight of a subsample comprising 15 randomly selected leaves. The random selection and number of leaves were considered adequate due to the morphological homogeneity of the leaves within each sample, despite the differences in plant height and diameter. The area was quantified using WinFolia Pro 2009 (Regent Instruments, Quebec, CA, US). The stem cross-sectional area-to-leaf area ratio (S_A_/L_A_) was measured for each plant as the cross section of the main stem base divided by LA. This variable was a proxy of the Huber value, as the relationship between sapwood area and stem cross-sectional area in a subsample of 11 plants was strong (*R*^2^ = 0.998) and had a slope close to one (*y* = 0.94 x – 0.061).

### Anatomical measurements

A 5-cm thick basal stem and root segment per sample was stored in FAA (formaldehyde–acetic acid–70% ethanol; 5:5:90, v:v:v) during a week and subsequently in 70% ethanol for anatomical analysis. Root segments were collected only in *Fagus*. Xylem cross sections 20–30 µm thick were cut using a sliding microtome (Leica SM 2400). The sections were stained with a solution of 1% safranine and 1% alcian blue following standard protocols (Rodríguez-García et al. 2014), dehydrated with 80%, 90%, 95% and 100% ethanol during 15 min in each solution, then placed into xylene and, finally, mounted with Eukitt for optical microscopy following standard protocols (Venturas et al. 2013). Images were obtained using a digital camera (Canon EOS 1000D, Japan) attached to a light microscope (Olympus BX51, Japan) for further image analysis with ImageJ (Schneider et al. 2012).

Anatomical variables were measured in 2017, 2018 and 2019 growth rings. For each ring, we calculated theoretical specific hydraulic conductance (k_sth_) of stems (Sk_sth_) and roots (Rk_sth_) based on the Hagen–Poiseuille equation of Tyree and Ewers (1991) as

$$k_{sth} = \frac{{\frac{\pi \rho }{{128\eta }}\mathop \sum \nolimits_{i = 1}^{n} \left( {D_{i}^{4} } \right)}}{S},$$

Where $\rho$ is the water density, $\eta$ is the water viscosity, $S$ is the sapwood cross-sectional area and D is the equivalent circular vessel diameter in at least 100 vessels.

### Data analysis

A linear mixed effect model (LMM) with browsing, tree species, date and their two- and three-way interactions as fixed factors (and with tree as a random factor), was used to assess their effects on physiological and biochemical variables. “Tree” was included within the random effect structure to account for repeated measurements on the same tree. When date was significant at *P* < 0.05, pairwise comparisons among dates were analyzed with Tukey HSD tests. The morphological variable S_A_/L_A_ and the shoot anatomical variables, sampled just once during the study, were compared between browsing treatments and species with two-way linear models. The rest of the morphological measurements (R/S and RD) and the root anatomical measurements, available only for unearthed *Fagus* saplings, were compared between browsing treatments with one-way linear models (see Table S1 available as Online Resource).

Backward stepwise selection was applied for all models through a series of simplifications to the minimal adequate model, following Crawley (2007). Data were log_10_-transformed to achieve the requirements of normality and homoscedasticity of residuals, when necessary; in these cases, back-transformed model estimates are reported in the text. The S_A_/L_A_ corresponding to one plant (95 × 10^–4^ cm^2^ cm^−2^) was removed from the data set, because it exceeded 10 times the limit value defined as an extreme outlier, i.e., three times the interquartile range. Furthermore, we run a Principal Component Analysis (PCA) after standardizing the study variables to separate observations along a multivariate plane. A subset of 10 study variables (only those showing a significant treatment effect in previous univariate comparisons: g_s_, A_mass_, LMA, [N], π_100_, π_0_, D_W_/T_W_, ε_max_, S_A_/L_A_, and SB) were included in the PCA.

Statistical analyses were carried out in R version 4.1.2 (R Development Core Team 2021) using the package nlme (Pinheiro et al. 2021) to perform linear mixed models, and the package stats (version 4.1.2) to perform linear models, calculate *P* values and run the PCA. Figures were produced using the package ggplot2 (Wickham 2016). All values presented in the text are means ± standard error.

## Results

Twelve years of contrasting, heavy ungulate browsing modified plant biomass distribution (i.e., R/S), plant hydraulic architecture (i.e., S_A_/L_A_), water status of leaves (i.e.,Ψ_pd_) and leaf gas exchange. This effect did not vary across dates (*P* value of treatment by date effect > 0.05 for all variables except Ψ_pd_) and was remarkably similar in *Fagus* and *Ilex* (*P* value of species by treatment effect > 0.05 for all variables), despite their different leaf habit and ecophysiology (Table 1; see Discussion and Online Resource Table S2). However, the studied variables were described and presented graphically separating the species and treatment.

**Table 1:** Statistical significance (*P* value) of main and interaction effects of treatment (**T**: Browsed and Unbrowsed), species (**S**) and, if applicable, date (**D**) during 2018 and 2019. **A** Linear mixed effect models (LMMs) for physiological and biochemical variables measured three times per year. **B** LMMs for pressure–volume variables measured once per year. **C** LMMs for biomass and anatomical variables, measured once at the end of the study. NS means non-significant effect (*P* > 0.05)

(A)							
Response variable	Treatment (T)	Species (S)	Date (D)	T:S	T:D	S:D	T:S:D
Ψ_pd_	**0.0199**	**0.0243**	** < 0.0001**	NS	**0.0026**	** < 0.0001**	NS
Ψ_md_	NS	** < 0.0001**	** < 0.0001**	NS	NS	NS	NS
A_area_	NS	**0.0295**	**0.0009**	NS	NS	NS	NS
g_s_	**0.0465**	**0.0012**	** < 0.0001**	NS	NS	**0.0013**	NS
C_i_	NS	NS	** < 0.0001**	NS	NS	NS	NS
ETR	NS	NS	** < 0.0001**	NS	NS	**0.0347**	NS
iWUE	NS	NS	** < 0.0001**	NS	NS	NS	NS
LMA	**0.0498**	** < 0.0001**	** < 0.0001**	NS	NS	** < 0.0001**	NS
A_mass_	**0.0372**	** < 0.0001**	**0.0011**	NS	NS	** < 0.0001**	NS
[C]	NS	NS	NS	NS	NS	NS	NS
[N]	**0.0142**	** < 0.0001**	**0.0069**	NS	NS	**0.0054**	NS
δ^13^C	NS	**0.0015**	** < 0.0001**	NS	NS	NS	NS
δ^15^N	NS	NS	** < 0.0001**	NS	NS	**0.0008**	NS

(B)							
Response variable	Treatment (T)	Species (S)	Date (D)	T:S	T:D	S:D	T:S:D
RWC	NS	NS	NS	NS	NS	NS	NS
R_s_	NS	NS	NS	NS	NS	NS	NS
R_a_	NS	NS	NS	NS	NS	NS	NS
π_100_	**0.0019**	NS	NS	NS	NS	NS	NS
π_0_	**0.0035**	NS	NS	NS	NS	NS	NS
D_W_/T_W_	**0.0232**	** < 0.0001**	**0.0078**	NS	NS	NS	NS
ε_max_	**0.0176**	NS	NS	NS	NS	NS	NS

(C)			
Response variable	Treatment (T)	Species (S)	T:S
S_A_/L_A_	**0.0313**	** < 0.0001**	NS
Sk_sth17_	NS	** < 0.0001**	NS
Sk_sth18_	NS	**0.0002**	NS
Sk_sth19_	NS	** < 0.0001**	NS
R/S	**0.0069**	–	–
RD	NS	–	–
RB	**0.0493**	–	–
SB	**0.0028**	–	–
Rk_sth17_	NS	–	–
Rk_sth18_	NS	–	–
Rk_sth19_	NS	–	–

*Ψ*_*pd*_ Predawn leaf water potential (MPa), *Ψ*_*md*_ Midday leaf water potential (MPa), *A*_*area*_ net photosynthetic rate (µmol m^−2^ s^−1^), *g*_*s*_ stomatal conductance of water vapor (mol m^−2^ s^−1^), *C*_*i*_ intercellular CO_2_ concentration (ppm), *ETR* electronic transport rate (μmol m^−2^ s^−1^), *iWUE* intrinsic water-use efficiency (mol mol^−1^), *LMA* leaf mass per area (g m^−2^), *A*_*mass*_ net photosynthetic rate per unit of leaf dry mass (nmol g^−1^ s^−1^), *[C]* carbon concentration (mg g^−1^), *[N]* nitrogen concentration (mg g^−1^), *δ*^*13*^*C* carbon isotope composition (‰), *δ*^*15*^*N* nitrogen isotope composition (‰), *RWC* relative water content at the turgor loss point (%), *R*_*s*_ relative symplastic water contents (%), *R*_*a*_ relative apoplastic water contents (%), *π*_*100*_ osmotic potential at full turgor (MPa), *π*_*0*_ osmotic potential at the turgor loss point (MPa), *D*_*W*_*/T*_*W*_ dry weight to turgor weight ratio (g g^−1^), *ε*_*max*_ maximum bulk modulus of elasticity (MPa), *S*_*A*_*/L*_*A*_ Stem cross-sectional area leaf area ratio (cm^2^ cm^−2^), *LA* total leaf area (cm^2^), Sk_sth17_, Sk_sth18_, Sk_sth19_ 2017, 2018 and 2019 stem theoretical specific hydraulic conductance (kg m^−1^ s^−1^ MPa^−1^), *R/S* root to shoot ratio (g g^−1^), *RD* root depth (cm), *RB* root dry biomass (g), *SB* shoot dry biomass (g), *Rk*_*sth17*_*, Rk*_*sth18*_*, Rk*_*sth19*_ 2017, 2018 and 2019 root theoretical specific hydraulic conductance (kg m^−1^ s^−1^ MPa^−1^)

These results were for 2 years of near average climate conditions. Precipitation and temperature during the 2018 study period (125 mm and 16.8 °C, respectively, over June through September) were similar to those of the 1994–2021 period. However, in the same period of 2019, precipitation was 13% above average (148 mm). It is noteworthy that the 2019 late summer measurement was preceded by 12.9 mm precipitation in the previous 48 h (see Figure S1 available as Online Resource).

### Leaf water potential

**Fig. 1:**
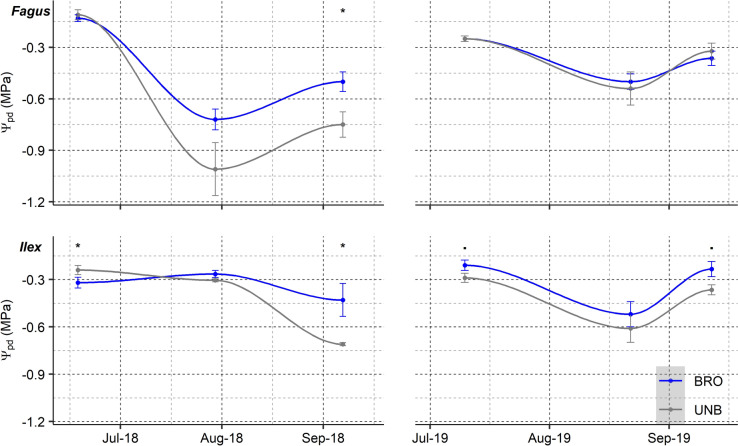
Evolution of predawn leaf water potential (Ψ_pd_) during the growing seasons of 2018 and 2019 in *Fagus* and *Ilex* saplings. *BRO* browsed, *UNB* unbrowsed. Means ± SE. *: *P* value < 0.05. **▪**: *P* value < 0.1

Significant differences in Ψ_pd_ were found between tree species, dates and ungulate exclosure treatments (Table 1A, Fig. 1). *Fagus* and *Ilex* plants subjected to sustained browsing showed 0.2–0.3 MPa higher Ψ_pd_ than unbrowsed plants in late summer 2018. *Ilex* plants showed the same trend in late spring and late summer 2019 (*P* < 0.1). On the contrary, Ψ_md_ was not affected by browsing. Ψ_md_ was significantly higher for *Ilex* than *Fagus* (*Ilex*: − 1.04 ± 0.04 vs. *Fagus*: − 1.72 ± 0.04 MPa; *P* < 0.0001) and varied across dates (*P* < 0.0001).

### Photosynthetic parameters

**Fig. 2:**
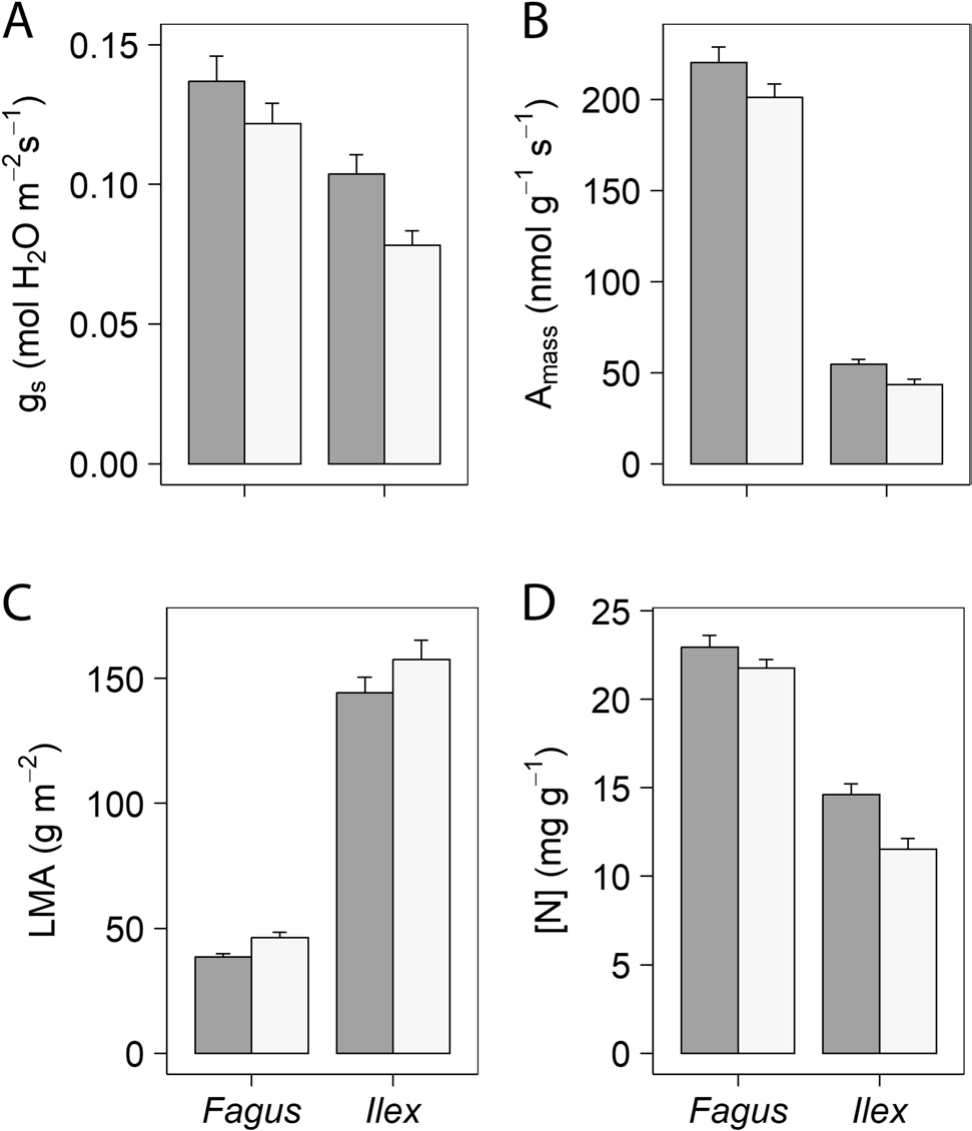
Photosynthetic and biochemical parameters significantly affected by sustained herbivory in *Fagus* and *Ilex* saplings across dates (see Table 1A). **A** Stomatal conductance of water vapor; **B** photosynthesis per-unit mass; **C** leaf mass per area; **D** nitrogen concentration. Closed bars correspond to browsed plants and open bars to unbrowsed plants. Means + SE

Browsed plants exhibited lower LMA (91.4 ± 7.6 vs. 102.0 ± 8.2 g m^−2^; *P* < 0.05), and higher rates of A_mass_ (137.4 ± 11.7 vs. 122.3 ± 11.0 nmol g^−1^ s^−1^; *P* < 0.05) and g_s_ (0.12 ± 0.01 vs. 0.10 ± 0.01 mol m^−2^ s^−1^; *P* < 0.05) than unbrowsed plants (Fig. 2). Browsing did not affect the rest of photosynthetic variables, which were remarkably different between species. For example, LMA in *Ilex* was more than three times higher than that of *Fagus* (150.99 ± 38.7 vs. 42.4 ± 1.3 g m^−2^; *P* < 0.0001), while A_mass_ and g_s_ were lower in *Ilex* than in *Fagus* (A_mass_: 49.1 ± 2.1 vs. 210.6 ± 5.9 nmol g^−1^ s^−1^; *P* < 0.0001. g_s_: 0.091 ± 0.005 vs. 0.129 ± 0.006 mol m^−2^ s^−1^; *P* < 0.01). The photosynthetic rate per unit leaf area (A_area_) was also significantly lower in *Ilex* than in *Fagus* (7.02 ± 0.27 vs. 8.81 ± 0.29 µmol m^−2^ s^−1^; *P* < 0.05; Table 1A). All photosynthetic variables exhibited significant variation over time (*P* < 0.01).

### Biochemical measurements

Browsed plants had 13% higher [N] than unbrowsed ones (18.79 ± 0.70 vs. 16.65 ± 0.77 mg g^−1^; *P* < 0.05) (Fig. 2). Leaf [C], and leaf δ^13^C and δ^15^N were not affected by sustained browsing. By species, *Ilex* leaves showed lower [N] (13.08 ± 0.47 vs. 22.36 ± 0.42 mg g^−1^; *P* < 0.0001) and higher δ^13^C (− 30.15 ± 0.13 vs. − 31.43 ± 0.15 ‰; *P* < 0.01) than *Fagus* leaves (Table 1A). The influence of date was statistically significant for all biochemical measurements except for [C] (*P* < 0.01).

### Pressure–volume curves

**Fig. 3:**
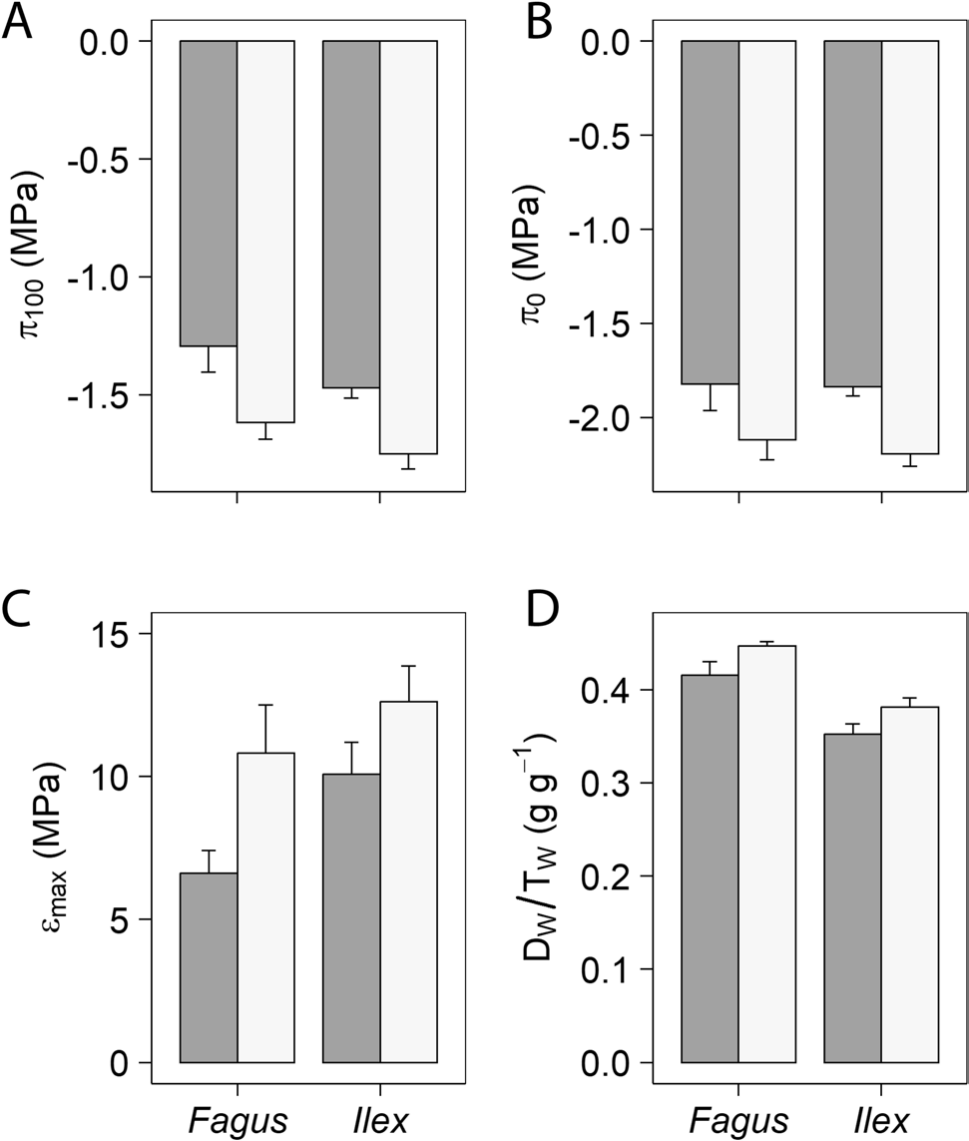
Pressure–volume curves variables significantly affected by sustained herbivory in *Fagus* and *Ilex* saplings across dates (see Table 1B). **A** Osmotic potential at full turgor; **B** osmotic potential at the turgor loss point; **C** maximum bulk modulus of elasticity;** D** dry weight-to-turgor weight ratio. Closed bars correspond to browsed plants and open bars to unbrowsed plants. Means + SE

Browsing had a significant effect on π_100_, π_0,_ ε_max_ and D_W_/T_W_ (Table 1B). Browsed plants had higher values of π_100_ and π_0_ and lower of ε_max_ and D_W_/T_W_ (Fig. 3). Species and date were only significant for D_W_/T_W_; this variable was higher in *Ilex* than in *Fagus* (0.43 ± 0.01 vs. 0.37 ± 0.01 g g^−1^; *P* < 0.05) and lower in 2018 than in the wetter 2019 (0.39 ± 0.01 vs. 0.41 ± 0.01 g g^−1^; *P* < 0.05) (Table 1B).

### Biomass partitioning

**Fig. 4:**
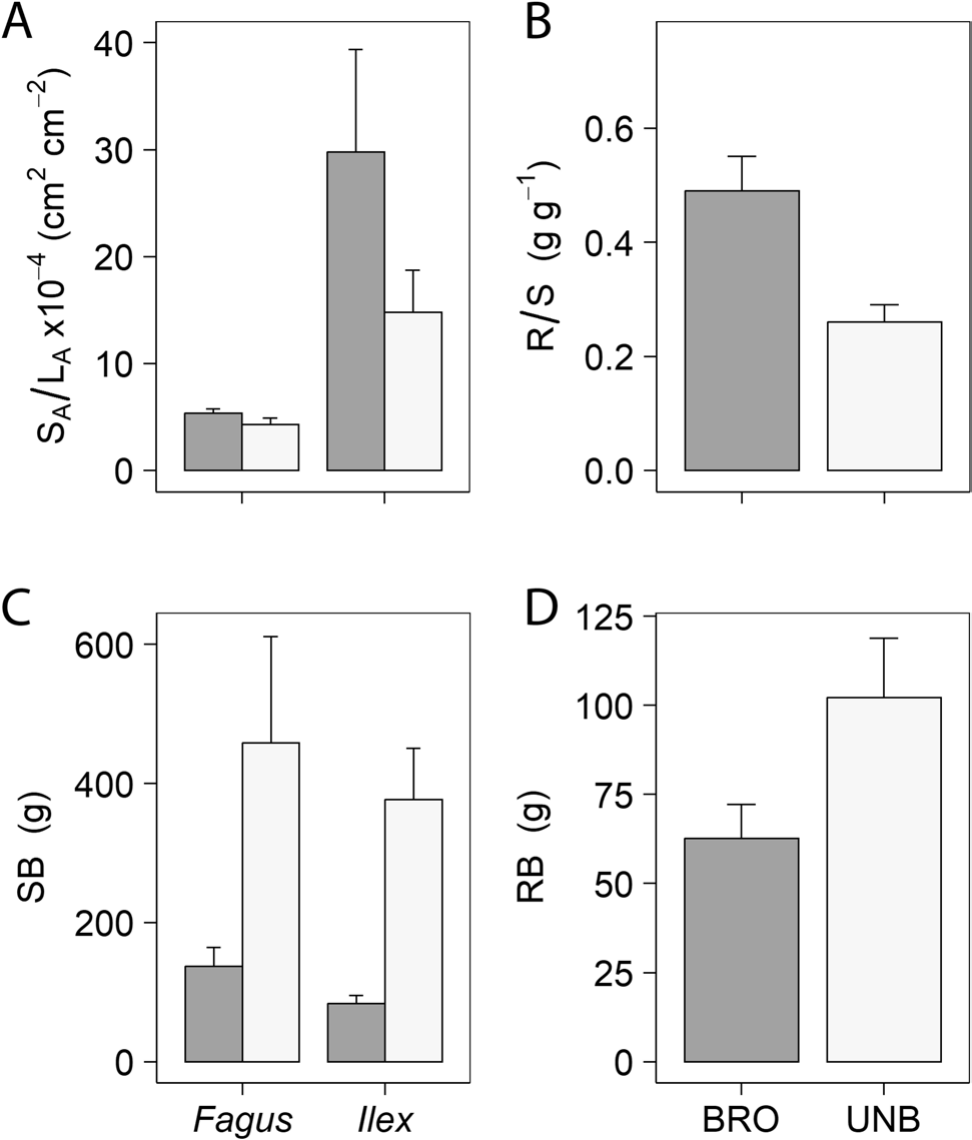
Biomass partitioning. Stem cross-sectional area-to-leaf area ratio (**A**), root-to-shoot ratio (**B**), and shoot and root dry mass (**C**, **D**, respectively) in browsed (closed bars) and unbrowsed (open bars) plants. Because the excavation of the root system of *Ilex* was too laborious and imprecise, **B** and **D** were measured only for *Fagus*. Means + SE

Plant biomass distribution was affected by sustained browsing. The variables R/S and RB for browsed *Fagus* plants were 88% higher and 33% lower than that for unbrowsed plants, respectively (R/S: 0.49 ± 0.06 vs. 0.26 ± 0.03 g g^−1^; RB: 68.7 ± 9.6 vs. 102.1 ± 16.8 g; *P* < 0.01). Shoot biomass (SB) were significantly lower in browsed plants compared to unbrowsed plants (113.2 ± 53.4 vs. 417.6 ± 257.1 g; *P* < 0.05). However, RD was not significantly different between browsed and unbrowsed plants (67.2 ± 12.4 vs. 69.7 ± 6.6 cm). The S_A_/L_A_ estimated at the whole-plant level increased with long-term browsing (18.9 10^–4^ ± 6,6 10^–4^ vs. 9.6 10^–4^ ± 2.6 10^–4^ cm^2^ cm^−2^; *P* < 0.05), and it was significantly higher in *Ilex* than in *Fagus* (22.3 10^–4^ ± 5.5 10^–4^ vs. 4.8 10^–4^ ± 0.4 10^–4^ cm^2^ cm^−2^; *P* < 0.0001) (Fig. 4; Table 1C).

### Anatomical measurements

Browsing had no significant effect on stem and root theoretical specific hydraulic conductance in 2017, 2018 and 2019 growth rings. The variable Sk_sth_ for *Fagus* plants was always higher than that for *Ilex* plants (*P* < 0.01) in 3 years analyzed (Table 1C).

**Fig. 5:**
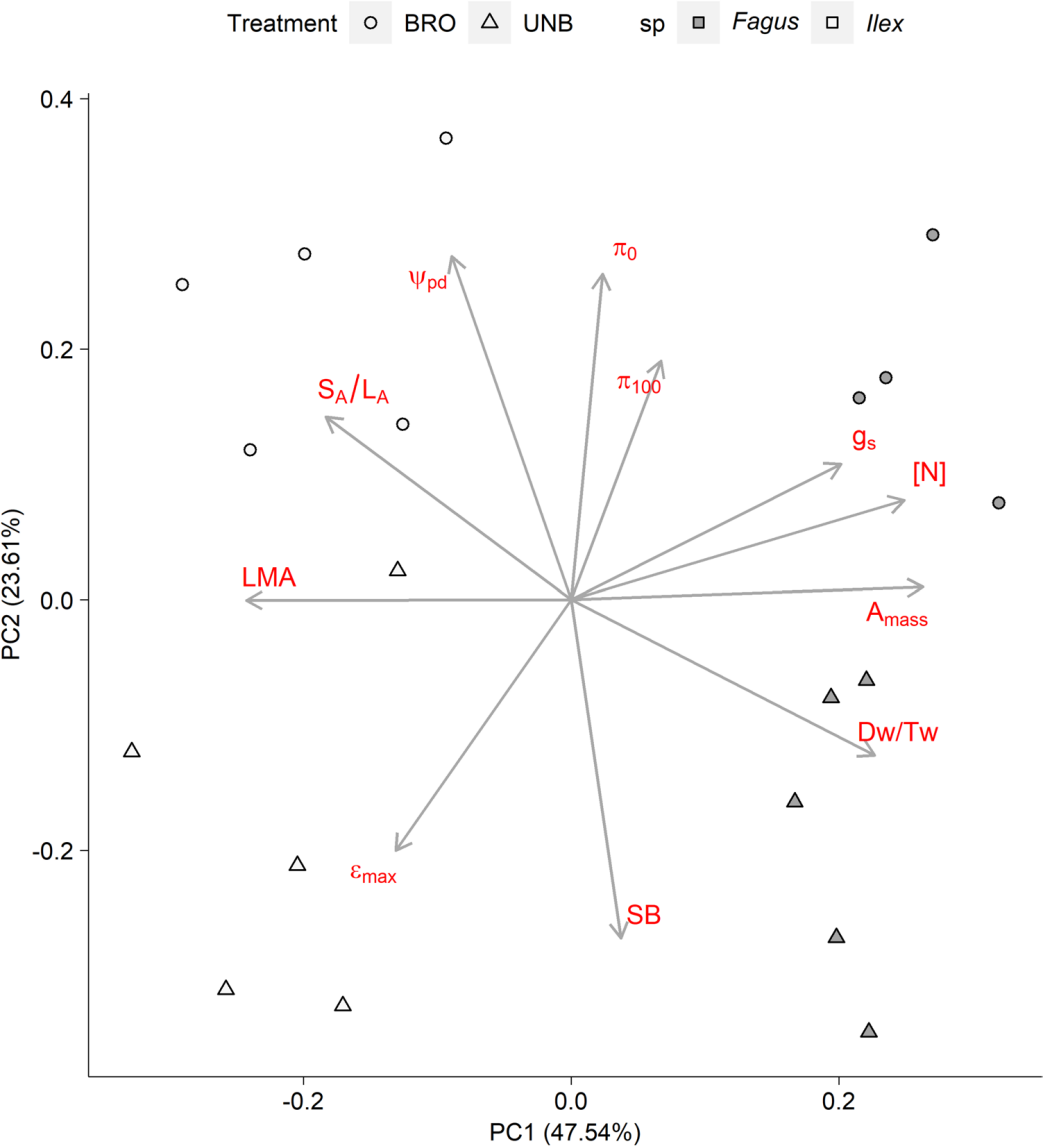
Principal component analysis results from a subset of measured variables. Dots represent plants, and arrows are proportional to the weight of variables in each principal component 1 and 2. *g*_*s*_ stomatal conductance of water vapor, *A*_*mass*_ photosynthesis per-unit mass, *LMA* leaf mass per area, *[N]* nitrogen concentration, *π*_*100*_ osmotic potential at full turgor, *π*_*0*_ osmotic potential at the turgor loss point, *D*_*W*_*/T*_*W*_ dry weight-to-turgor weight ratio, ε_max_ maximum bulk modulus of elasticity, *S*_*A*_*/L*_*A*_ stem cross-sectional area-to-leaf area ratio, *SB* shoot dry mass, *Ψ*_*pd*_ predawn leaf water potential, *BRO* browsed, *UNB* unbrowsed

To synthesize previous results, we ran a PCA with a subset of 10 variables showing a significant treatment effect in previous univariate comparisons (g_s_, A_mass_, LMA, N, π_100_, π_0_, D_W_/T_W_, ε_max_, S_A_/L_A_ and SB). The first two principal components (PC) explained > 70% of the variance. The PC1 separated the species *Fagus* and *Ilex* by their contrasting resource-use leaf strategy, whereas the PC2 separated the treatments by the contrasting sclerophylly and drought tolerance of browsed vs. unbrowsed plants (Fig. 5).

## Discussion

The legacy of herbivory in two contrasting species has modulated a specific pattern of biomass partitioning, morphology, physiology and architecture that makes browsed plants more sensitive to further browsing and concurrent drought. These findings have implications for the regeneration of these and similar species in environments where herbivory and drought co-occur.

Repeated shoot browsing by ungulates resulted in plants with lower above- and below-ground biomass. The loss of root biomass may be related to at least two interrelated processes. On the one hand, reduced whole plant C gain due to foliage removal limits root growth, and may cause plants to allocate resources to shoot tissue regeneration, at the expense of their investment in root biomass (Smith and Schowalter 2001). On the other hand, the disruption of hormonal signals above ground, such as increased jasmonic acid concentration or reduced auxins after browsing (Erb et al. 2009), can lead to a reorganisation in root metabolism and architecture, reducing root size over time (Wasternack 2007). However, as it was hypothesized, although browsing reduced root biomass, the R/S of browsed plants was higher than that of unbrowsed plants. The depth reached by the taproot was not significantly different between treatments, suggesting a preferential root inversion in soil foraging for water, the main abiotic limiting factor during the summer season (Padilla and Pugnaire 2007). Long-lasting effects of browsing on hydraulic architecture extended to the higher sapwood-to-leaf area ratio in browsed plants, estimated by the S_A_/L_A_ at the whole plant level. This variable may be directly and indirectly related to herbivory: directly, due to the mechanical removal of shoots, and indirectly due to the smaller leaf area of browsed plants (mean total leaf area 3111.0 ± 815.2 vs. 10,743.0 ± 2619.3 cm^2^, *P* < 0.05, data not shown). Notably, browsing did not trigger any change in xylem anatomy, supporting that a change in the xylem conducting area-to-leaf area ratio is the main factor favoring water supply to leaves in browsed plants (Pittermann et al. 2014; Leonard et al. 2024).

The plant architectural changes that long-term herbivory has generated in browsed plants have likely influenced organ-level physiology and biochemistry. Leaves of browsed plants showed a higher [N] than those of unbrowsed plants. This is inconsistent with the lower root biomass of browsed plants, which would limit soil nitrogen uptake (McMurtrie et al. 2012; Postma et al. 2014; Kiba and Krapp 2016), assuming equal nutritional specialization of fine roots of all plants (Hodge 2004; Göransson et al. 2007; da Silva et al. 2011). However, a higher R/S ratio in browsed plants may improve the distribution of N above ground. Lower leaf biomass of browsed plants results in lower dilution of assimilated N per unit leaf mass compared to unbrowsed plants. Moreover, the higher photosynthetic rate per unit mass of leaves in browsed plants suggests a higher N assimilation capacity (Hikosaka and Osone 2009). The higher foliar [N] may be associated with a greater capacity to synthesize defensive compounds (phenols in *Fagus* and phenols and alkaloids in *Ilex*), but it also makes leaves more attractive to ungulates.

Similarly, herbivory legacy on root and shoot biomass partitioning, specifically R/S, rather than root depth, affected leaf predawn water status (Boyer 1995). The browsed plants had a higher Ψ_pd_ than the unbrowsed ones at the end of summer 2018, a year climatically similar to 1994–2021 period (see Figure S1 available as Online Resource). This higher water status at dawn in the browsed saplings suggests a greater ability to avoid water stress compared to unbrowsed saplings; it would partly compensate the loss of plant foliage by buffering the negative impact of summer drought on leaf carbon fixing capacity (Aranda et al. 2002; Robson et al. 2009). The dwarfing of the plant body caused by ungulates reflected in high R/S and S_A_/L_A_, together with similar k_sth_, favor the recovery of Ψ once the stomata close. In fact, the Ψ_md_ was not affected by herbivory, indicating that a higher Ψ gradient developed throughout the day in browsed plants. Although in some studies a lower xylem water transport capacity has been found in plants subjected to recurrent herbivory (Pittermann et al. 2014), our results showed that the theoretical hydraulic conductivity was independent of the herbivory legacy. Since irradiance (i.e., global site factor) was also similar in plants inside and outside the ungulate exclusion fence, we argue that leaf-level water expenditure in plants subjected to sustained herbivory was higher than in unbrowsed plants. The higher stomatal conductance observed in the leaves of browsed plants supports this view. The higher Ψ_pd_, S_A_/L_A_ and foliar [N] of browsed saplings favor CO_2_ assimilation, and explain the higher A_mass_ observed in browsed plants. However, the comparatively thinner leaves produced by browsed plants, as suggested by their lower LMA compared to those of unbrowsed plants, explains why A_area_ was similar for browsed and unbrowsed plants.

The analysis of PV curves suggested that, in relation to their higher Ψ_pd_, browsed plants also had lower need or capacity to tolerate drought; lower need due to lower water stress or lower capacity due to lower ability to osmoregulate. Tolerance to water stress is related to the osmotic and elastic adjustments defined by π_100_ and ε_max_, respectively (Dreyer et al. 1990; Zobel 1996). Leaves formed by plants under long-term herbivory were characterised by higher π_100_ and π_0_, and lower ε_max_ and D_w_/T_w_ values than those developed under the fence protection. Although osmotic adjustment was not explicitly quantified by measuring π_100_ on dates without water stress, our results suggest that browsed saplings accumulate solutes less actively than unbrowsed saplings, and thus are less able to tolerate drought stress. First, at a given level of water potential, the higher values of π_100_ and π_0_ in leaves of browsed plants imply a lower capacity to maintain cell turgor. Second, the higher elasticity of their cell wall, determined by ε_max_, leads to a lower capacity to maintain or recover cell volume (Corcuera et al. 2002). Third, the lower D_w_/T_w_ typical of low-LMA leaves with thin cell walls (Hassiotou et al. 2010) suggests a lower capacity of cells to withstand low turgor loss point values (Nardini 2022). This lower tolerance to dehydration of leaves in browsed plants could aggravate the response to drought in drier years, as both species show a limited capacity to osmoregulate in the understory, where shade limits mechanisms of drought tolerance (Aranda et al. 2001; Rodríguez-Calcerrada et al. 2010). In line with these results, Ueda et al. (2024) showed higher xylem vulnerability to cavitation in severely defoliated *Fagus crenata* plants than in non-defoliated ones.

Taking all these results together, we observe that sustained herbivory has modulated leaf traits towards the fast-returning end of the leaf economics spectrum (Wright et al. 2004). Thus, herbivory legacy resulted in a lower degree of sclerophylly, reflected in lower LMA and D_w_/T_w_, higher [N] and A_mass_, higher π_100_ and π_0_, and lower ε_max_, a syndrome resembling that of short-lived leaves with an acquisitive strategy of rapid return of nutrients invested in plant biomass (Corcuera et al. 2002; Wright et al. 2004). This herbivory-induced modification of the leaf economics spectrum is in line with the positive plant–herbivore feedback phenomenon (Craig 2010). This phenomenon evidences that browsed plants are more prone to suffer subsequent herbivory due to compensatory growth being associated with reduced herbivory defence (du Toit et al. 1990; Barrere et al. 2022). To counteract biomass losses, the plant invests nutrients in forming acquisitive organs conducing to rapid growth (Edenius et al. 1993), which may lead to increased attractiveness to ungulates, due to higher leaf nitrogen content (Wright et al. 2004) and digestibility (Cornelissen et al. 2004), associated among other traits with lower LMA (Baraza et al. 2010). The modification of plant architecture produced by ungulates, which allows a high number of shoots to be maintained at the optimum browsing height, may also favor this positive feedback loop (Hartley et al. 1997). Contrary to our results, shorter-term studies on (insect) herbivory have described opposite changes in damaged tree saplings towards a more conservative resource-use strategy (Peschiutta et al. 2018), with this highlighting the need to contextualize herbivory effects on stress resistance according to the type and duration of herbivory. Interestingly, although *Ilex* and *Fagus* belong to the opposite end of the leaf economic spectrum axis (Tomás Marín et al. 2023; Jacobs et al. 2022; Niinemets et al. 2003), and present different strategies against ungulate pressure (Obeso 1997; Svendsen 2001; Scarnati et al. 2009) and summer drought (Aranda et al. 2008; Valladares and Niinemets 2008), both species responded similarly to herbivory, suggesting that constitutive differences in leaf herbivory resistance between species do not modulate plasticity to severe, long-term browsing. This similar long-term response of *Ilex* and *Fagus* to herbivory does not suggest a differential impact of browsing on the relative abundance of both species.

### Concluding statements

We have compared tree saplings growing in an exclosure fence for 12 years with saplings growing outside, fenced individually for 2 years of the study (i.e., after herbivory has subsided). This simple experimental approach, which has never been applied before (to the best of our knowledge), allows studying herbivory legacy, without the confounding, uncontrolled effects of current-year herbivory. We conclude that sustained herbivory has long- (at least 2 years) lasting legacy effects on plant architecture and biomass partitioning, which modifies the leaves towards more resource-acquisitive, less stress tolerant phenotypes. Since plasticity in leaf water relations is related to the intensity of the experienced stress, higher hydraulic recovery capacity (i.e.,Ψ_pd_) in historically browsed plants may have lowered the need (and cost) to phenotypically adjust leaf properties, such as osmotic contents. On the other hand, C limitations caused by recurrent foliage removal and need to resprout may reduce the capacity to accumulate solutes, and favor less C costly elastic adjustments.

## Supplementary Information

Below is the link to the electronic supplementary material.

Supplementary file1 (PDF 15 KB)

Supplementary file2 (PDF 77 KB)

Supplementary file3 (PDF 64 KB)

## Data Availability

The data sets used and/or analysed during the current study are available from the corresponding author on reasonable request.

## References

[CR1] Acevedo P, Ferreres J, Jaroso R, Durán M, Escudero MA, Marco J, Gortázar C (2010) Estimating roe deer abundance from pellet group counts in Spain: an assessment of methods suitable for Mediterranean woodlands. Ecol Indic 10(6):1226–1230. 10.1016/j.ecolind.2010.04.006

[CR2] Acevedo P, Quirós-Fernández F, Casal J, Vicente J (2014) Spatial distribution of wild boar population abundance: basic information for spatial epidemiology and wildlife management. Ecol Indic 36:594–600. 10.1016/j.ecolind.2013.09.019

[CR3] Aragon S, Braza F, San Jose C (1995) Socioeconomic, physiognomic, and climatic factors determining the distribution pattern of roe deer *Capreolus capreolus* in Spain. Acta Theriol (Warsz) 40(1):37–43. 10.4098/AT.arch.95-4

[CR5] Aranda I, Gil L, Pardos J (2001) Effects of thinning in a *Pinus sylvestris* L. stand on foliar water relations of *Fagus sylvatica* L. seedlings planted within the pinewood. Trees-Struct Funct 15(6):358–364. 10.1007/s004680100109

[CR6] Aranda I, Gil L, Pardos JA (2002) Physiological responses of *Fagus sylvatica* L. seedlings under *Pinus sylvestris* L. and Quercus pyrenaica Willd. overstories. Forest Ecol Manag 162(2–3):153–164. 10.1016/S0378-1127(01)00502-3

[CR7] Aranda I, Robson TM, Rodríguez-Calcerrada J, Valladares F (2008) Limited capacity to cope with excessive light in the open and with seasonal drought in the shade in Mediterranean *Ilex aquifolium* populations. Trees-Struct Funct 22(3):375–384. 10.1007/s00468-007-0192-5

[CR8] Bansal S (2015) The interactive effects of drought and Herbivory on Ecophysiology of trees. In: Mahalingam R (ed) Combined Stresses in Plants: Physiological, Molecular, and Biochemical Aspects. Springer International Publishing, Switzerland, pp 245–259

[CR9] Bansal S, Hallsby G, Löfvenius MO, Nilsson MC (2013) Synergistic, additive and antagonistic impacts of drought and herbivory on *Pinus sylvestris*: leaf, tissue and whole-plant responses and recovery. Tree Physiol 33(5):451–463. 10.1093/treephys/tpt019 23525156 10.1093/treephys/tpt019

[CR10] Baraza E, Zamora R, Hódar JA (2010) Species-specific responses of tree saplings to herbivory in contrasting light environments: an experimental approach. Ecoscience 17(2):156–165. 10.2980/17-2-3286

[CR11] Barrere J, Collet C, Saïd S, Bastianelli D, Verheyden H, Courtines H, Bonnet A, Segrestin J, Boulanger V (2022) Do trait responses to simulated browsing in *Quercus robur* saplings affect their attractiveness to *Capreolus capreolus* the following year? Environ Exp Bot 194:104743. 10.1016/j.envexpbot.2021.104743

[CR12] Blundell AG, Peart DR (2001) Growth strategies of a shade-tolerant tropical tree: the interactive effects of canopy gaps and simulated herbivory. J Ecol 89(4):608–615. 10.1046/j.0022-0477.2001.00581.x

[CR13] Boyer JS (1995) Measuring the Water Status of Plants and Soils. Academic Press, San Diego

[CR14] Burbaite L, Csányi S (2009) Roe deer population and harvest changes in Europe. Est J EcoL. 10.3176/eco.2009.3.02

[CR15] Carpio AJ, Guerrero-Casado J, Barasona JA, Tortosa FS (2017) Ecological impacts of wild ungulate overabundance on Mediterranean Basin ecosystems. In: Menéndez A, Sands N (eds) Ungulates: Evolution, Diversity and Ecology. Nova Science Pub Inc, New York, pp 111–157

[CR16] Carpio AJ, Apollonio M, Acevedo P (2021) Wild ungulate overabundance in Europe: contexts, causes, monitoring and management recommendations. Mamm Rev 51(1):95–108. 10.1111/mam.12221

[CR17] Carvajal Acosta AN, Agrawal AA, Mooney K (2023) Plant water-use strategies as mediators of herbivore drought response: ecophysiology, host plant quality and functional traits. J Ecol 111(3):687–700. 10.1111/1365-2745.14059

[CR18] Corcuera L, Camarero JJ, Gil-Pelegrín E (2002) Functional groups in *Quercus* species derived from the analysis of pressure-volume curves. Trees-Struct Funct 16(7):465–472. 10.1007/s00468-002-0187-1

[CR19] Cornelissen JHC, Quested HM, Gwynn-Jones D, Van Logtestijn RSP, De Beus MAH, Kondratchuk A, Callaghan TV, Aerts R (2004) Leaf digestibility and litter decomposability are related in a wide range of subarctic plant species and types. Funct Ecol 18(6):779–786. 10.1111/j.0269-8463.2004.00900.x

[CR20] Côté SD, Rooney TP, Tremblay JP, Dussault C, Waller DM (2004) Ecological impacts of deer overabundance. Annu Rev Ecol Evol Syst 35:113–147. 10.1146/annurev.ecolsys.35.021103.105725

[CR21] Craig TP (2010) The resource regulation hypothesis and positive feedback loops in plant-herbivore interactions. Popul Ecol 52(4):461–473. 10.1007/s10144-010-0210-0

[CR22] Crawley MJ (2007) The R book. Wiley, Hoboken

[CR23] da Silva EV, Bouillet JP, De Moraes Gonçalves JL, Junior CHA, Trivelin PCO, Hinsinger P, Jourdan C, Nouvellon Y, Stape JL, Laclau JP (2011) Functional specialization of *Eucalyptus* fine roots: contrasting potential uptake rates for nitrogen, potassium and calcium tracers at varying soil depths. Funct Ecol 25(5):996–1006. 10.1111/j.1365-2435.2011.01867.x

[CR24] de Tomás MS, Galán Díaz J, Rodríguez-Calcerrada J, Prieto I, de la Riva EG (2023) Linking functional composition moments of the sub-Mediterranean ecotone with environmental drivers. Front Plant Sci 14:1303022. 10.3389/fpls.2023.1303022 38143583 10.3389/fpls.2023.1303022 PMC10748396

[CR25] Didion M, Kupferschmid AD, Bugmann H (2009) Long-term effects of ungulate browsing on forest composition and structure. Forest Ecol Manag 258:S44–S55. 10.1016/j.foreco.2009.06.006

[CR26] Drexhage M, Colin F (2003) Effects of browsing on shoots and roots of naturally regenerated sessile oak seedlings. Ann for Sci 60(2):173–178. 10.1051/forest:2003010

[CR27] Dreyer E, Bousquet F, Ducrey M (1990) Use of pressure volume curves in water relation analysis on woody shoots: influence of rehydration and comparison of four European oak species. Ann Sci Forest 47(4):285–297. 10.1051/forest:19900401

[CR28] du Toit JT, Bryant JP, Frisby K (1990) Regrowth and palatability of Acacia shoots following pruning by African savanna browsers. Ecology 71(1):149–154. 10.2307/1940255

[CR29] Edenius L, Danell K, Bergström R, Bergstrom R (1993) Impact of herbivory and competition on compensatory growth in woody plants: winter browsing by moose on scots pine. Oikos 66(2):286–292. 10.2307/3544816

[CR30] Erb M, Lenk C, Degenhardt J, Turlings TC (2009) The underestimated role of roots in defense against leaf attackers. Trends Plant Sci 14(12):653–659. 10.1016/j.tplants.2009.08.006 19736036 10.1016/j.tplants.2009.08.006

[CR31] Farooq M, Hussain M, Wahid A, Siddique K (2012) Drought stress in plants: an overview. In: Aroca R (ed) Plant Responses to Drought Stress: From Morphological to Molecular Features. Springer, Berlin, pp 1–33

[CR32] Flexas J, Escalona JM, Medrano H (1999) Water stress induces different levels of photosynthesis and electron transport rate regulation in grapevines. Plant Cell Environ 22(1):39–48. 10.1046/j.1365-3040.1999.00371.x

[CR33] Frerker K, Sabo A, Waller D (2014) Long-term regional shifts in plant community composition are largely explained by local deer impact experiments. PLoS One 9(12):e115843. 10.1371/journal.pone.0115843 25551827 10.1371/journal.pone.0115843 PMC4281217

[CR34] Gely C, Laurance SGW, Stork NE (2020) How do herbivorous insects respond to drought stress in trees? Biol Rev 95(2):434–448. 10.1111/brv.12571 31750622 10.1111/brv.12571

[CR35] Gieger T, Thomas FM (2002) Effects of defoliation and drought stress on biomass partitioning and water relations of *Quercus robur* and *Quercus petraea*. Basic Appl Ecol 3(2):171–181. 10.1078/1439-1791-00091

[CR36] Gill RMA, Beardall V (2001) The impact of deer on woodlands: the effects of browsing and seed dispersal on vegetation structure and composition. Forestry 74(3):209–218. 10.1093/forestry/74.3.209

[CR37] Göransson H, Fransson AM, Jönsson-Belyazid U (2007) Do oaks have different strategies for uptake of N, K and P depending on soil depth? Plant Soil 297(1):119–125. 10.1007/s11104-007-9325-2

[CR38] Gossner MM, Pašalić E, Lange M, Lange P, Boch S, Hessenmöller D, Müller J, Socher SA, Fischer M, Schulze ED, Weisser W (2014) Differential responses of herbivores and herbivory to management in temperate European beech. PLoS One 9(8):e104876. 10.1371/journal.pone.0104876 25119984 10.1371/journal.pone.0104876 PMC4132021

[CR39] Gutbrodt B, Mody K, Dorn S (2011) Drought changes plant chemistry and causes contrasting responses in lepidopteran herbivores. Oikos 120(11):1732–1740. 10.1111/j.1600-0706.2011.19558.x

[CR40] Hammond WM, Williams AP, Abatzoglou JT, Adams HD, Klein T, López R, Sáenz-Romero C, Hartmann H, Breshears DD, Allen CD (2022) Global field observations of tree die-off reveal hotter-drought fingerprint for Earth’s forests. Nat Commun 13(1):1761. 10.1038/s41467-022-29289-2 35383157 10.1038/s41467-022-29289-2 PMC8983702

[CR41] Hanley ME, Lamont BB, Fairbanks MM, Rafferty CM (2007) Plant structural traits and their role in anti-herbivore defence. Perspect Plant Ecol 8(4):157–178. 10.1016/j.ppees.2007.01.001

[CR42] Hartley SE, Iason GR, Duncan AJ, Hitchcock D (1997) Feeding behaviour of red deer (*Cervus elaphus*) offered Sitka Spruce saplings (*Picea sitchensis*) grown under different light and nutrient regimes. Funct Ecol 11(3):348–357. 10.1046/j.1365-2435.1997.00094.x

[CR43] Hassiotou F, Renton M, Ludwig M, Evans JR, Veneklaas EJ (2010) Photosynthesis at an extreme end of the leaf trait spectrum: how does it relate to high leaf dry mass per area and associated structural parameters? J Exp Bot 61(11):3015–3028. 10.1093/jxb/erq128 20484320 10.1093/jxb/erq128 PMC2892145

[CR44] Hikosaka K, Osone Y (2009) A paradox of leaf-trait convergence: why is leaf nitrogen concentration higher in species with higher photosynthetic capacity? J Plant Res 122(3):245–251. 10.1007/s10265-009-0222-z 19252965 10.1007/s10265-009-0222-z

[CR45] Hodge A (2004) The plastic plant: root responses to heterogeneous supplies of nutrients. New Phytol 162(1):9–24. 10.1111/j.1469-8137.2004.01015.x

[CR46] Huberty AF, Denno RF (2004) Plant water stress and its consequences for herbivorous insects: a new synthesis. Ecology 85(5):1383–1398. 10.1890/03-0352

[CR47] Jacobs K, Jonard M, Muys B, Ponette Q (2022) Shifts in dominance and complementarity between sessile oak and beech along ecological gradients. J Ecol 110(10):2404–2417. 10.1111/1365-2745.13958

[CR48] Johnston DB, Cooper DJ, Hobbs NT (2007) Elk browsing increases aboveground growth of water-stressed willows by modifying plant architecture. Oecologia 154(3):467–478. 10.1007/s00442-007-0854-4 17934763 10.1007/s00442-007-0854-4

[CR49] Kiba T, Krapp A (2016) Plant nitrogen acquisition under low availability: regulation of uptake and root architecture. Plant Cell Physiol 57(4):707–714. 10.1093/pcp/pcw052 27025887 10.1093/pcp/pcw052 PMC4836452

[CR50] Lecomte X, Bugalho MN, Catry FX, Fernandes PM, Cera A, Caldeira MC (2024) Ungulates mitigate the effects of drought and shrub encroachment on the fire hazard of Mediterranean oak woodlands. Ecol Appl. 10.1002/eap.2971 38581136 10.1002/eap.2971

[CR51] Leland Russell F, Zippin DB, Fowler NL (2001) Effects of white-tailed deer (*Odocoileus virginianus*) on plants, plant populations and communities: a review. Am Midl Nat 146(1):1–26. 10.1674/0003-0031(2001)146[0001:EOWTDO]2.0.CO;2

[CR52] Leonard HE, Ciambrone M, Pittermann J (2024) Species-specific responses drive browsing impacts on physiological and functional traits in *Quercus agrifolia* and *Umbellularia californica*. PLoS One 19(7):e0287160. 10.1371/journal.pone.0287160 39047008 10.1371/journal.pone.0287160 PMC11268663

[CR53] Leuschner C (2020) Drought response of European beech (*Fagus sylvatica* L.)—a review. Perspect Plant Ecol 47:125576. 10.1016/j.ppees.2020.125576

[CR54] López-Sánchez A, Peláez M, Dirzo R, Fernandes GW, Seminatore M, Perea R (2019) Spatio-temporal variation of biotic and abiotic stress agents determines seedling survival in assisted oak regeneration. J Appl Ecol 56(12):2663–2674. 10.1111/1365-2664.13500

[CR55] Malamy JE (2005) Intrinsic and environmental response pathways that regulate root system architecture. Plant Cell Environ 28(1):67–77. 10.1111/j.1365-3040.2005.01306.x 16021787 10.1111/j.1365-3040.2005.01306.x

[CR56] Maxwell K, Johnson GN (2000) Chlorophyll fluorescence—a practical guide. J Exp Bot 51(345):659–668. 10.1093/jxb/51.345.659 10938857 10.1093/jxb/51.345.659

[CR57] McMurtrie RE, Iversen CM, Dewar RC, Medlyn BE, Näsholm T, Pepper DA, Norby RJ (2012) Plant root distributions and nitrogen uptake predicted by a hypothesis of optimal root foraging. Ecol Evol 2(6):1235–1250. 10.1002/ece3.266 22833797 10.1002/ece3.266 PMC3402197

[CR58] Morales-Molino C, Tinner W, Perea R, Carrión JS, Colombaroli D, Valbuena-Carabaña M, Zafra E, Gil L (2019) Unprecedented herbivory threatens rear-edge populations of *Betula* in southwestern Eurasia. Ecology 100(11):e02833. 10.1002/ecy.2833 31323116 10.1002/ecy.2833

[CR59] Mountford EP, Peterken GF (2003) Long-term change and implications for the management of wood-pastures: experience over 40 years from Denny Wood. New Forest Forestry 76(1):19–43. 10.1093/forestry/76.1.19

[CR60] Munns R (1988) Why measure osmotic adjustment? Aust J Plant Physiol 15(6):717–726. 10.1071/PP9880717

[CR61] Nardini A (2022) Hard and tough: the coordination between leaf mechanical resistance and drought tolerance. Flora 288:152023. 10.1016/j.flora.2022.152023

[CR62] Niinemets Ü, Valladares F (2006) Tolerance to shade, drought, and waterlogging of temperate northern hemisphere trees and shrubs. Ecol Monogr 76(4):521–547. 10.1890/0012-9615(2006)076

[CR63] Niinemets Ü, Valladares F, Ceulemans R (2003) Leaf-level phenotypic variability and plasticity of invasive *Rhododendron ponticum* and non-invasive *Ilex aquifolium* co-occurring at two contrasting European sites. Plant Cell Environ 26(6):941–956. 10.1046/j.1365-3040.2003.01027.x 12803621 10.1046/j.1365-3040.2003.01027.x

[CR64] Nuttle T, Yerger EH, Stoleson SH, Ristau TE (2011) Legacy of top-down herbivore pressure ricochets back up multiple trophic levels in forest canopies over 30 years. Ecosphere 2(1):1–11. 10.1890/ES10-00108.1

[CR65] Obeso JR (1997) The induction of spinescence in European holly leaves by browsing ungulates. Plant Ecol 129(2):149–156. 10.1023/A:1009767931817

[CR66] Padilla FM, Pugnaire FI (2007) Rooting depth and soil moisture control Mediterranean woody seedling survival during drought. Funct Ecol 21(3):489–495. 10.1111/j.1365-2435.2007.01267.x

[CR67] Peláez M, López-Sánchez A, Rodríguez-Calcerrada J, Dirzo R, Perea R (2025) Responses of oak seedlings to increased herbivory and drought: a possible trade-off?. Ann Bot 135(1-2):341–356. 10.1093/aob/mcae178 39383257 10.1093/aob/mcae178 PMC11805927

[CR68] Pellerin M, Saïd S, Richard E, Hamann JL, Dubois-Coli C, Hum P (2010) Impact of deer on temperate forest vegetation and woody debris as protection of forest regeneration against browsing. Forest Ecol Manag 260(4):429–437. 10.1016/j.foreco.2010.04.031

[CR69] Pendergast TH, Hanlon SM, Long ZM, Royo AA, Carson WP (2015) The legacy of deer overabundance: long-term delays in herbaceous understory recovery. Can J Forest Res 46(3):362–369. 10.1139/cjfr-2015-0280

[CR70] Perea R, Girardello M, San Miguel A (2014) Big game or big loss? High deer densities are threatening woody plant diversity and vegetation dynamics. Biodivers Conserv 23(5):1303–1318. 10.1007/s10531-014-0666-x

[CR71] Perea R, López-Sánchez A, Pallarés J, Gordaliza GG, González-Doncel I, Gil L, Rodríguez-Calcerrada J (2020) Tree recruitment in a drought- and herbivory-stressed oak-beech forest: implications for future species coexistence. Forest Ecol Manag 477:118489. 10.1016/j.foreco.2020.118489

[CR72] Peschiutta ML, Scholz FG, Goldstein G, Bucci SJ (2018) Herbivory alters plant carbon assimilation, patterns of biomass allocation and nitrogen use efficiency. Acta Oecol 86:9–16. 10.1016/j.actao.2017.11.007

[CR73] Pinheiro J, Bates D, DebRoy S, Sarkar D, R Core Team (2021) nlme: Linear and nonlinear mixed effects models; [accessed 2024 Apr 13]. https://cran.r-project.org/web/packages/nlme/nlme.pdf

[CR74] Pittermann J, Lance J, Poster L, Baer A, Fox LR (2014) Heavy browsing affects the hydraulic capacity of *Ceanothus rigidus* (Rhamnaceae). Oecologia 175(3):801–810. 10.1007/s00442-014-2947-1 24817157 10.1007/s00442-014-2947-1

[CR75] Poorter L, Markesteijn L (2008) Seedling traits determine drought tolerance of tropical tree species. Biotropica 40(3):321–331. 10.1111/j.1744-7429.2007.00380.x

[CR76] Postma JA, Dathe A, Lynch JP (2014) The optimal lateral root branching density for maize depends on nitrogen and phosphorus availability. Plant Physiol 166(2):590–602. 10.1104/pp.113.233916 24850860 10.1104/pp.113.233916 PMC4213091

[CR77] Pulido FJ, Díaz M (2005) Regeneration of a Mediterranean oak: a whole-cycle approach. Ecoscience 12(1):92–102. 10.2980/i1195-6860-12-1-92.1

[CR78] Rennenberg H, Loreto F, Polle A, Brilli F, Fares S, Beniwal RS, Gessler A (2006) Physiological responses of forest trees to heat and drought. Plant Biol 8(5):556–571. 10.1055/s-2006-924084 16773557 10.1055/s-2006-924084

[CR79] Retuerto R, Fernández-Lema B, Obeso JR (2006) Changes in photochemical efficiency in response to herbivory and experimental defoliation in the dioecious tree *Ilex aquifolium*. Int J Plant Sci 167(2):279–289. 10.1086/498919

[CR80] Rita A, Cherubini P, Leonardi S, Todaro L, Borghetti M (2015) Functional adjustments of xylem anatomy to climatic variability: insights from long-term *Ilex aquifolium* tree-ring series. Tree Physiol 35(8):817–828. 10.1093/treephys/tpv055 26142450 10.1093/treephys/tpv055

[CR81] Robichaux RH (1984) Variation in the tissue water relations of two sympatric Hawaiian *Dubautia* species and their natural hybrid. Oecologia 65(1):75–81. 10.1007/BF00384465 28312112 10.1007/BF00384465

[CR82] Robson TM, Rodríguez-Calcerrada J, Sánchez-Gómez D, Aranda I (2009) Summer drought impedes beech seedling performance more in a sub-Mediterranean forest understory than in small gaps. Tree Physiol 29(2):249–259. 10.1093/treephys/tpn023 19203950 10.1093/treephys/tpn023

[CR83] Rodríguez-Calcerrada J, Pardos JA, Aranda I (2010) Contrasting responses facing peak drought in seedlings of two co-occurring oak species. Forestry 83(4):369–378. 10.1093/forestry/cpq019

[CR84] Rodríguez-Calcerrada J, Salomón RL, Gordaliza GG, Miranda JC, Miranda E, De La Riva EG, Gil L (2019) Respiratory costs of producing and maintaining stem biomass in eight co-occurring tree species. Tree Physiol 39(11):369–378. 10.1093/treephys/tpz069 10.1093/treephys/tpz069 31211374

[CR85] Rodríguez-García A, López R, Martín JA, Pinillos F, Gil L (2014) Resin yield in *Pinus pinaster* is related to tree dendrometry, stand density and tapping-induced systemic changes in xylem anatomy. Forest Ecol Manag 313:47–54. 10.1016/j.foreco.2013.10.038

[CR86] Rooney TP, Waller DM (2003) Direct and indirect effects of white-tailed deer in forest ecosystems. Forest Ecol Manag 181(1–2):165–176. 10.1016/S0378-1127(03)00130-0

[CR87] Rozas V, Camarero JJ, Sangüesa-Barreda G, Souto M, García-González I (2015) Summer drought and ENSO-related cloudiness distinctly drive *Fagus sylvatica* growth near the species rear-edge in northern Spain. Agric Forest Meteorol 201:153–164. 10.1016/j.agrformet.2014.11.012

[CR88] Salleo S, Nardini A, Gullo MAL (1997) Is sclerophylly of Mediterranean evergreens an adaptation to drought? New Phytol 135(4):603–612. 10.1046/j.1469-8137.1997.00696.x

[CR89] Scarnati L, Attorre F, de Sanctis M, Farcomeni A, Francesconi F, Mancini M, Bruno F (2009) A multiple approach for the evaluation of the spatial distribution and dynamics of a forest habitat: the case of Apennine beech forests with *Taxus baccata* and *Ilex aquifolium*. Biodivers Conserv 18(12):3099–3113. 10.1007/s10531-009-9629-z

[CR90] Schneider CA, Rasband WS, Eliceiri KW (2012) NIH Image to ImageJ: 25 years of image analysis. Nat Methods 9(7):671–675. 10.1038/nmeth.2089 22930834 10.1038/nmeth.2089 PMC5554542

[CR91] Scholander PF, Bradstreet ED, Hemmingsen EA, Hammel HT (1965) Sap pressure in vascular plants: negative hydrostatic pressure can be measured in plants. Science 148(3668):339–346. 10.1126/science.148.3668.339 17832103 10.1126/science.148.3668.339

[CR92] Smith JP, Schowalter TD (2001) Aphid-induced reduction of shoot and root growth in Douglas-fir seedlings. Ecol Entomol 26(4):411–416. 10.1046/j.1365-2311.2001.00335.x

[CR93] Staver AC, Bond WJ (2014) Is there a “browse trap”? Dynamics of herbivore impacts on trees and grasses in an African savanna. J Ecol 102(3):595–602. 10.1111/1365-2745.12230

[CR94] Strauss SY, Agrawal AA (1999) The ecology and evolution of plant tolerance to herbivory. Trends Ecol Evol 14(5):179–185. 10.1016/S0169-5347(98)01576-6 10322530 10.1016/s0169-5347(98)01576-6

[CR95] Sun X, Sun Y, Cao X, Zhai X, Callaway RM, Wan J, Luke S, Huang W, Ding J (2023) Trade-offs in non-native plant herbivore defences enhance performance. Ecol Lett 26:1584–1596. 10.1111/ele.14283 37387416 10.1111/ele.14283

[CR96] Svendsen CR (2001) Effects of marcescent leaves on winter browsing by large herbivores in northern temperate deciduous forests. Alces-N Am 37(2):475–483

[CR97] Taiz L, Zeiger E (2010) Plant Physiology, 5th edn. Sinauer Associates Inc., Suderland

[CR98] Tellería JL, Virgós E (1997) Distribution of an increasing roe deer population in a fragmented Mediterranean landscape. Ecography 20(3):247–252. 10.1111/j.1600-0587.1997.tb00368.x

[CR99] Tiffin P (2000) Mechanisms of tolerance to herbivore damage: what do we know? Evol Ecol 14(4–6):523–536. 10.1023/A:1010881317261

[CR100] Turner NC (1988) Measurement of plant water status by the pressure chamber technique. Irrigation Sci 9(4):289–308. 10.1007/BF00296704

[CR101] Tyree MT, Ewers FW (1991) The hydraulic architecture of trees and other woody plants. New Phytol 119(3):345–360. 10.1111/j.1469-8137.1991.tb00035.x

[CR102] Ueda M, Izumi K, Ueo S (2024) Repeated artificial defoliation soon after full leaf expansion, simulating insect damage, reduces xylem hydraulic transport safety in Japanese beech (*Fagus crenata* Blume). Trees 38(2):303–313. 10.1007/s00468-023-02475-5

[CR103] Valente AM, Acevedo P, Figueiredo AM, Fonseca C, Torres RT (2020) Overabundant wild ungulate populations in Europe: management with consideration of socio-ecological consequences. Mamm Rev 50(4):353–366. 10.1111/mam.12202

[CR104] Valladares F, Niinemets Ü (2008) Shade tolerance, a key plant feature of complex nature and consequences. Annu Rev Ecol Evol S 39:237–257. 10.1146/annurev.ecolsys.39.110707.173506

[CR105] van Hees AFM (1997) Growth and morphology of pedunculate oak (*Quercus robur* L) and beech (*Fagus sylvatica* L) seedlings in relation to shading and drought. Ann Sci Forest 54(1):9–18. 10.1051/forest:19970102

[CR106] Venturas M, López R, Gascó A, Gil L (2013) Hydraulic properties of European elms: xylem safety-efficiency tradeoff and species distribution in the Iberian Peninsula. Trees 27:1691–1701. 10.1007/s00468-013-0916-7

[CR107] Wasternack C (2007) Jasmonates: an update on biosynthesis, signal transduction and action in plant stress response, growth and development. Ann Bot 100(4):681–697. 10.1093/aob/mcm079 17513307 10.1093/aob/mcm079 PMC2749622

[CR108] White TCR (1984) The abundance of invertebrate herbivores in relation to the availability of nitrogen in stressed food plants. Oecologia 63(1):90–105. 10.1007/BF00379790 28311171 10.1007/BF00379790

[CR109] Wickham H (2016) ggplot2: Elegant Graphics for Data Analysis, 2nd edn. Springer International Publishing, Switzerland

[CR110] Wright IJ, Reich PB, Westoby M, Ackerly DD, Baruch Z, Bongers F, Cavender-Bares J, Chapin T, Cornellssen JHC, Diemer M, Flexas J, Garnier E, Groom P, Gulias J, Hikosaka K, Lamont B, Lee T, Lee W, Lusk C, Midgley J, Navas ML, Niinemets Ü, Oleksyn J, Osada N, Poorter H, Poot P, Prior L, Pyankov V, Roumet C, Thomas S, Tjoelker M, Veneklaas E, Villar R (2004) The worldwide leaf economics spectrum. Nature 428(6985):821–827. 10.1038/nature02403 15103368 10.1038/nature02403

[CR111] Zamora R, Gómez JM, Hódar JA, Castro J, García D (2001) Effect of browsing by ungulates on sapling growth of Scots pine in a mediterranean environment: consequences for forest regeneration. Forest Ecol Manag 144(1–3):33–42. 10.1016/S0378-1127(00)00362-5

[CR112] Zandalinas SI, Mittler R, Balfagón D, Arbona V, Gómez-Cadenas A (2018) Plant adaptations to the combination of drought and high temperatures. Physiol Plant Pathol 162(1):2–12. 10.1111/ppl.12540 10.1111/ppl.12540 28042678

[CR113] Zobel DB (1996) Variation of water relations parameters with extended rehydration time, leaf form, season, and proportion of leaf. Can J Forest Research 26(2):175–185. 10.1139/x26-021

