# Supplementary material for: Herbivory legacy modifies leaf economic spectrum and drought tolerance in two tree species
Source: Oecologia. 2025 Feb 26;207(2):39. doi: 10.1007/s00442-025-05678-4 (PMC11865174; doi:10.1007/s00442-025-05678-4)
Supplement: Supplementary file 1 — Supplementary file1 (PDF 15 KB) [file 442_2025_5678_MOESM1_ESM.pdf]

Table S1: Statistical analyses by response variable and associated predictors depending on measurement date. **T**: Treatment (Browsed and Unbrowsed); **S**: Species (*Ilex* and *Fagus*); **D**: Date (2018, 2019; LSP, MSU, LSU). **LSP**: Late spring. **MSU**: Mid-summer. **LSU**: Late summer. **S_A/L_A**: stem cross-sectional area to leaf area ratio. **SB**: shoot dry biomass. **R/S**: root-shoot ratio. **RD**: root depth. **RB**: root dry biomass.

**Response variable**	**Statistical analyses**	**Predictors**	**2018**			**2019**		
			**LSP**	**MSU**	**LSU**	**LSP**	**MSU**	**LSU**
**Leaf water potential**	Linear mixed effect model	T (x2) S (x2) D (x6)	X	X	X	X	X	X
**Photosynthetic parameters**	Linear mixed effect model	T (x2) S (x2) D (x6)	X	X	X	X	X	X
**Biochemical measurements**	Linear mixed effect model	T (x2) S (x2) D (x6)	X	X	X	X	X	X
**Pressure-volume curves**	Linear mixed effect model	T (x2) S (x2) D (x2)			X			X
**Biomass (S_A/L_A, SB) and shoot anatomical measurements**	Two-way linear model	T (x2) S (x2)						X
**Biomass (R/S, RD, RB) and root anatomical measurements**	One-way linear model	T (x2)						X
