# Supplementary material for: Herbivory legacy modifies leaf economic spectrum and drought tolerance in two tree species
Source: Oecologia. 2025 Feb 26;207(2):39. doi: 10.1007/s00442-025-05678-4 (PMC11865174; doi:10.1007/s00442-025-05678-4)
Supplement: Supplementary file 2 — Supplementary file2 (PDF 77 KB) [file 442_2025_5678_MOESM2_ESM.pdf]

Table S2: Means + SE of the variables studied, by species and treatment. **BRO**: Browsed; **UNB**: Unbrowsed.[1]

Variable	Units	*Fagus*						*Ilex*					
		BRO			UNB			BRO			UNB		
Ψ_{pd}	MPa	-0.41	±	0.04	-0.50	±	0.07	-0.33	±	0.03	-0.42	±	0.04
Ψ_{md}	MPa	-1.68	±	0.07	-1.76	±	0.05	-1.03	±	0.06	-1.04	±	0.05
A_{area}	µmol m^{-2} s^{-1}	8.59	±	0.50	9.03	±	0.28	7.71	±	0.40	6.33	±	0.31
g_s	mol m^{-2} s^{-1}	0.137	±	0.009	0.122	±	0.007	0.104	±	0.007	0.078	±	0.005
C_i	ppm	277.68	±	5.09	254.92	±	5.67	256.00	±	5.03	249.91	±	4.46
ETR	µmol m^{-2} s^{-1}	44.69	±	3.41	47.84	±	2.16	50.87	±	2.72	43.20	±	2.61
iWUE	mol mol^{-1}	64.84	±	3.08	79.14	±	3.52	79.20	±	3.23	83.83	±	2.79
LMA	g m^{-2}	38.4	±	1.3	46.3	±	2.1	144.3	±	6.3	157.7	±	7.7
A_{mass}	nmol g^{-1} s^{-1}	220.1	±	8.8	201.1	±	7.5	54.7	±	2.6	43.5	±	3.0
[C]	mg g^{-1}	458.31	±	7.28	466.51	±	4.35	473.87	±	4.16	470.06	±	3.04
[N]	mg g^{-1}	22.95	±	0.67	21.78	±	0.48	14.63	±	0.60	11.52	±	0.61
$\delta^{13}C$	‰	-31.74	±	0.24	-31.13	±	0.17	-30.38	±	0.14	-29.91	±	0.21
$\delta^{15}N$	‰	-0.99	±	0.06	-1.23	±	0.12	-1.33	±	0.15	-1.93	±	0.23
RWC	%	0.84	±	0.02	0.83	±	0.02	0.85	±	0.01	0.86	±	0.01
R_s	%	0.67	±	0.06	0.75	±	0.05	0.78	±	0.04	0.73	±	0.06
R_a	%	0.33	±	0.06	0.25	±	0.05	0.22	±	0.04	0.27	±	0.06
π_{100}	MPa	-1.29	±	0.11	-1.62	±	0.07	-1.47	±	0.04	-1.75	±	0.06

[1] **Ψ_{pd}**: Predawn leaf water potential; **Ψ_{md}**: Midday leaf water potential; **A_{area}**: net photosynthetic rate; **g_s**: stomatal conductance of water vapor; **C_i**: intercellular CO_2 concentration; **ETR**: electronic transport rate; **iWUE**: intrinsic water-use efficiency; **LMA**: leaf mass per area; Amass: net photosynthetic rate per unit of leaf dry mass; **[C]**: carbon concentration; **[N]**: nitrogen concentration; **$\delta^{13}C$**: carbon isotope composition; **$\delta^{15}N$**: nitrogen isotope composition; **RWC**: relative water content at the turgor loss point; **R_s**: relative symplastic water contents; **R_a**: relative apoplastic water contents; **π_{100}**: osmotic potential at full turgor ; **π_0**: osmotic potential at the turgor loss point; **D_W/T_W**: dry weight to turgor weight ratio; εmax: maximum bulk modulus of elasticity; **RD**: root depth; **RB**: root dry biomass; **SB**: shoot dry biomass; **R/S**: root to shoot ratio; **LA**: total leaf area; **S_A/L_A**: stem cross-sectional area to leaf area ratio; **S_{ksth17}**, **S_{ksth18}**, **S_{ksth19}**: 2017, 2018 and 2019 stem theoretical specific hydraulic conductance; **R_{ksth17}**, **R_{ksth18}**, **R_{ksth19}**: 2017, 2018 and 2019 root theoretical specific hydraulic conductance

π_0	MPa	-1.82	±	0.14	-2.12	±	0.11	-1.84	±	0.05	-2.20	±	0.07
D_W/T_W	g g^{-1}	0.42	±	0.01	0.45	±	0.01	0.35	±	0.01	0.38	±	0.01
ε_{max}	MPa	6.62	±	0.80	10.82	±	1.68	10.07	±	1.13	12.61	±	1.26
S_A/L_A	x10^{-4} cm^2 cm^{-2}	5.3	±	0.4	4.3	±	0.6	29.8	±	9.6	14.8	±	3.9
LA	cm^2	4816	±	1225	16522	±	3747	1406	±	190	4964	±	364
Sk_{sth17}	Kg m^{-1} s^{-1} MPa^{-1}	4.26	±	0.46	4.42	±	0.41	0.36	±	0.07	0.46	±	0.07
Sk_{sth18}	Kg m^{-1} s^{-1} MPa^{-1}	4.06	±	1.10	3.42	±	0.95	0.54	±	0.05	0.54	±	0.07
Sk_{sth19}	Kg m^{-1} s^{-1} MPa^{-1}	1.74	±	0.43	1.52	±	0.29	0.36	±	0.06	0.30	±	0.03
SB	g	137.07	±	26.99	458.38	±	153.08	83.31	±	12.21	376.91	±	73.96
RB	g	62.68	±	9.55	102.13	±	16.75	-	±	-	-	±	-
RD	cm	69.7	±	6.6	67.3	±	12.4	-	±	-	-	±	-
R:S	g g^{-1}	0.48	±	0.1	0.26	±	0.1	-	±	-	-	±	-
Rk_{sth17}	Kg m^{-1} s^{-1} MPa^{-1}	3.18	±	0.22	3.76	±	0.66	-	±	-	-	±	-
Rk_{sth18}	Kg m^{-1} s^{-1} MPa^{-1}	3.10	±	0.84	2.30	±	0.60	-	±	-	-	±	-
Rk_{sth19}	Kg m^{-1} s^{-1} MPa^{-1}	1.26	±	0.26	1.52	±	0.24	-	±	-	-	±	-
