# Supplementary material for: Herbivory legacy modifies leaf economic spectrum and drought tolerance in two tree species
Source: Oecologia. 2025 Feb 26;207(2):39. doi: 10.1007/s00442-025-05678-4 (PMC11865174; doi:10.1007/s00442-025-05678-4)

Figure S1: Precipitation (bars) and temperature (continuous line) in the 2018 **(A)** and 2019 **(B)** growing seasons. **P**: precipitation (mm), **T**: temperature (ºC). Red dashed lines mark the dates of sampling.

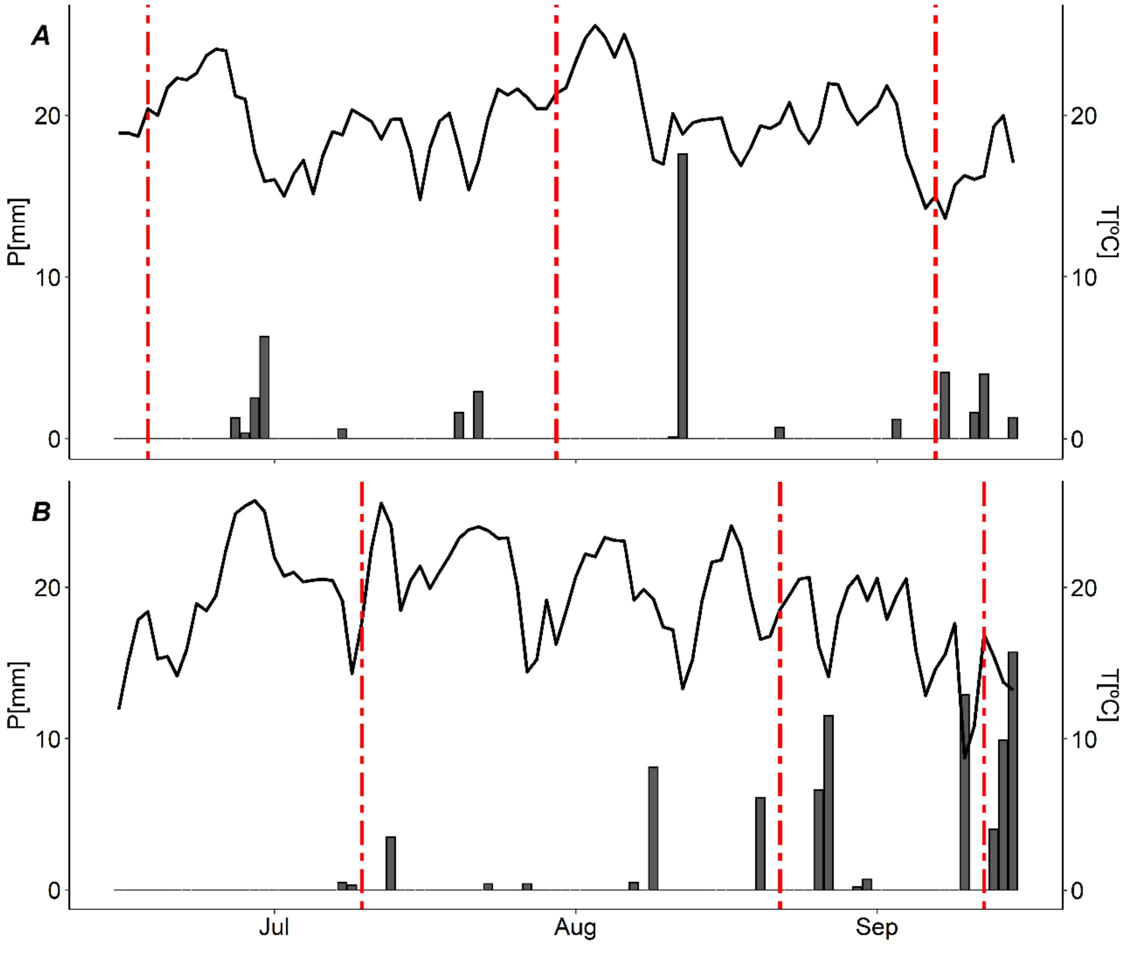

Supplement: Supplementary file 3 — Supplementary file3 (PDF 64 KB) [file 442_2025_5678_MOESM3_ESM.pdf]
